# Development of working memory, processing speed, and psychosocial functions in patients with pediatric cancer

**DOI:** 10.1038/s41390-024-03512-w

**Published:** 2024-08-29

**Authors:** Kirstin Schuerch, Saskia Salzmann, Leonie Steiner, Karen Lidzba, Andrea Klein, Jochen Roessler, Regula Everts

**Affiliations:** 1 Division of Neuropediatrics, Development and Rehabilitation, Department of Pediatrics, Inselspital, Bern University Hospital, Bern, Switzerland; 2 Division of Pediatric Hematology/Oncology, Department of Pediatrics, Inselspital, Bern University Hospital, Bern, Switzerland; 3 Graduate School for Health Science, University of Bern, Bern, Switzerland

## Abstract

**Abstract:**

Many patients after pediatric cancer suffer from long-term cognitive difficulties. This study investigates the development of cognitive and psychosocial functions between diagnosis and one year after cancer treatment and reveals insight into the association between cognitive and psychosocial development and various risk factors. This retrospective clinical record review included fifty-seven patients, aged 4–16 years, that were examined at the beginning of the cancer treatment (T1) and one year after cancer treatment (T2) to evaluate the development of working memory (WM), processing speed (PS), psychosocial functions, and quality of life (QoL). About half of the patients showed stable/favorable cognitive development (PS 51.9%; WM 41.4%). The other half exhibited a non-favorable cognitive development, with a decrease of performance between T1 and T2. In 51.6–77.4%, psychosocial functions remained stable/increased between T1 and T2 and QoL scores remained stable in 42.9–61.9%. Changes in prosocial behavior correlated with the development of PS (*r* = 0.472, *p* = 0.010). Age at T1 predicted PS at T2 (*p* = 0.020) and sex predicted peer relations at T2 (*p* = 0.046). About half of the patients showed stable/favorable whereas the other half experiencing non-favorable cognitive development. The observed disparities in initial and subsequent cognitive performances highlight the importance of early individualized patient monitoring and interventions.

**Impact:**

- We investigated the cognitive and psychosocial development of pediatric cancer patients between diagnosis and one year after termination of cancer treatment.
- About half of the patients showed stable or favorable cognitive development in processing speed and working memory.
- The other half exhibited a non-favorable cognitive development, with decreasing performance.
- Baseline working memory and processing speed was negatively correlated with the respective change score.
- Changes in prosocial behavior were positively correlated with the development of processing speed.
- Early individualized patient monitoring and intervention is of crucial importance after pediatric cancer and its treatment.

## Introduction

Long-term cognitive sequelae of cancer in childhood and adolescence often occur. However, around 60% of pediatric cancer survivors have favorable cognitive development.^1^ Studies on resilience suggest that the individual and the environment can potentially mitigate the effects of cancer and its treatment and therefore might function as protective factors.^2,3^ Siegwart et al. (2022)^3^ describe that the presence of protective factors such as cognitive and social resources was associated with cognitive functioning and quality of life (QoL) in pediatric cancer survivors years after termination of cancer treatment. Strong social and cognitive resources could therefore buffer adverse long-term effects of pediatric cancer.^3^

It is well known that cancer and its treatment is associated with long-term cognitive difficulties.^4–6^ Around 35% of a Swiss sample of pediatric cancer survivors present a cognitive performance of one or more standard deviation below the normative range.^7^ Deficits in working memory and processing speed are common regardless of the type of cancer and treatment.^8^ Working memory is the memory system that holds and manipulates information in short-term storage. It is central to nearly all daily tasks, and serves as a mental workspace that helps to continuously store and use information.^9^ Processing speed enables one to use cognitive processes effectively^10^ and refers to the speed at which various mental processes can be executed.^11^ Working memory and processing speed are closely linked to school success and build the foundation for many complex cognitive abilities, such as reading, mental arithmetic, and attention.^12^ Due to their slow and prolonged development, working memory and processing speed are particularly vulnerable to adverse effects such as cancer and its treatment.^13^ Consequently, children and adolescents with cancer need to recover from cancer on the one hand and on the other hand need to master the upcoming developmental steps. Because cancer might disrupt brain development and maturation,^5,8,14–16^ cancer and its treatment can interrupt developmental steps in an age-appropriate manner, leading to cognitive, social and behavioral difficulties even years after diagnosis.^17–19^

Findings in adult survivors of pediatric cancer suggest that cognitive problems may be related to psychosocial functions such as, i.e., emotional distress.^20^ Cognitive functioning is closely linked to social attainment and poor QoL in pediatric cancer survivors. In particular processing speed performance has been associated with unemployment and worse emotional and mental health.^20^ This highlights the need to not only focus on cognitive but also on psychosocial long-term outcome in patients after pediatric cancer.

Risk factors known to affect cognitive development negatively include among others young age at treatment, female sex, genetics, cancer type and location, chemotherapy dose, field size of radiation, and longer time since treatment.^4,5,15,21,22^ Greater cognitive difficulties are associated with a larger field size of radiation^23^ and higher radiation dose^24^ as well as intrathecal chemotherapy^25^ and high chemotherapy dose.^24^ However, a substantial proportion of pediatric patients with brain tumors (ranging from 20 to 50%), exhibit cognitive difficulties even before initiating cancer treatment.^26^

The balance between protective and risk factors might account for the variance in cognitive outcome.^27^ Research on protective factors suggests that both the individual (i.e., personal resources such as optimism) and the environment (i.e., social resources, disease education) can potentially mitigate the effects of risk factors such as cancer and its treatment.^28^ Protective resources become particularly noticeable when an individual faces critical life events. Therefore, protective factors differ from coping mechanisms used to manage everyday stressors.^2^ Risk and protective factors cannot be completely separated from each other. For instance, low socioeconomic status might be a risk factor for cognitive difficulties, whereas high socioeconomic status might function as a protective factor. However, little is known about how protective and risk factors are associated with the cognitive development after childhood cancer.

We therefore examined and compared the development of patient’s cognitive and psychosocial functions and QoL at the beginning and one year after termination of cancer treatment. In addition, we investigated how possible risk factors relate to cognitive and psychosocial development. As this study investigates retrospective clinical data to gain insight into the long-term development of cognitive functions, we refrain from the formulation of hypothesis. A deeper insight into cognitive development may help clinicians to promote individual therapeutic strategies that strengthen the maturational trajectory of young patients after pediatric cancer.

## Methods

We conducted a retrospective clinical record review on pediatric cancer patients treated at the Division of Pediatric Hematology and Oncology, Children’s Hospital in Bern, Switzerland from 2010 to 2020. The data were collected as part of neuropsychological routine examinations. After the introduction of the general consent at the Children’s University Hospital (2016), data were only used if the patients (or legal guardian) had signed (consented to) the general consent or had been informed of the right to object by means of the general consent. If a patient (or legal guardian) had not provided feedback in the form of consent or refusal after 30 days, it can be assumed that the patient (or legal guardian) has not exercised his or her right to object. Patients who rejected the general consent were not included in the study. Local ethics committee Bern approved the study (KEK-Nr. 2021-00342).

### Pediatric cancer patients

All children and adolescents who meet the following criteria were included: (a) treated for an oncological disease at the Children’s Hospital Bern between 1st of January 2010 and 31st of December 2020; (b) aged between 4 and 16 years; (c) available neuropsychological examination in the first two weeks of cancer treatment (T1) and one year after termination of cancer treatment (T2). Children and adolescents under 4 years and over 16 years of age were not assessed with neuropsychological tests at our clinic and were therefore excluded. Additionally, patients who did not complete the neuropsychological tests required for the present study across the two time points were excluded. In addition, patients with preexisting cognitive impairments or developmental disorders were excluded. Lastly, patients who participated in the Brainfit study^29,30^ during time point 1 and 2 were not included in the analyses. Fifty-seven patients were included in the analysis (25 boys, 32 girls; mean age (T1) = 9.44 years, SD = 2.92 years, range = 4.02–14.41 years). Because the data were collected retrospectively through records, there were missing data in some variables, resulting in different sample sizes (processing speed: *n* = 54; working memory: *n* = 41; psychosocial functions: *n* = 31; QoL: *n* = 21). The considerably smaller sample size in working memory is due to the restricted age range of the normative values of the working memory index, whereby scores are missing in children <6 years. Further, questionnaires to assess psychosocial functions were often not returned. At T1, nonverbal intelligence was assessed in *n* = 44 patients.

### Outcome measures

Age at assessment, sex, central nervous system (CNS) involvement of the tumor, cancer type, and treatment modality were collected from medical records. The standardized neuropsychological examinations performed in clinical practice during the first two weeks of cancer treatment (T1) and one year after termination of the cancer treatment (T2) included comprehensive neuropsychological tests and questionnaires.

At T1 nonverbal intelligence was assessed using the Test of Nonverbal Intelligence, third edition TONI-3; internal consistency: α = 0.89–0.97; test-retest reliability in the range of *r* = 0.90 (interval: one week).^31^

Processing speed was evaluated with the subtests symbol coding and symbol search of the German version of the Wechsler Preschool and Primary Scale of Intelligence, third edition (HAWIVA-III; reliability *r* = 0.76–0.90, for validity see test manual)^32^ for patients aged 4.0 to 5.11 years. The processing speed subtests coding and symbol search of the German version of the Wechsler Intelligence Scale for Children, fourth edition (HAWIK-IV; reliability *r* = 0.84–0.90; validity see test manual)^33^ were applied with patients aged 6.00 to 16.11 years. In the coding task, the patient draws as quickly as possible as many symbols that belong to the corresponding numbers using a key. In the symbol search subtest, the patient marks whether or not certain target symbols appear in a given row of symbols as quickly as possible. Both subtests are paper-pencil tests and are time-restricted to 120 s. In the symbol search test, the number of incorrect identified symbols is subtracted from the total number of symbols. In the coding test, all correct items are included in the total sum score. An index value for processing speed was calculated out of these two subtests according to the manuals for both age groups.

Working memory was evaluated in patients > 6 years using the working memory index value of the HAWIK-IV (reliability *r* = 0.89–0.93; validity see test manual).^33^ This index value is composed of two subtests namely digit span and letter-number sequencing. In the digit span subtest, a sequence of numbers was read out by the examiner. The number of digits increased with at least one of two correct enumerations. First, the patient repeats the digits in the same order, second, the patient tries to remember the order in which the digits are presented, but repeat them in reverse order. In the letter-number sequencing subtest, patients hear a sequence of letters and numbers and repeat the sequence by starting with the numbers in ascending order followed by the letters in alphabetical order.

Psychosocial functions were evaluated with the German parent-report version of the Strength and Difficulties Questionnaire.^34^ The SDQ is a screening questionnaire for behavioral problems of children and adolescents aged 4–17 years. It contains five domains: emotional problems, conduct problems, hyperactivity and inattention, peer problems, and prosocial behavior. Each domain consists of five items using a 3‐point Likert scale. For each domain, a sum score was computed (range 0–10), with higher scores indicating worse function, except for prosocial behavior, where higher scores indicate better functioning. Additionally, a total difficulties score was calculated by adding the values from the four scales referring to problems (without the scale for prosocial behavior; range 0–40). As in Mader and collegues,^35^ we applied German reference^36,37^ to categorize patients as normal, borderline, and abnormal for each scale. For the German version of the SDQ, the total difficulty score shows an internal reliability of α = 0.77 and the five subscales present a test–retest reliability of *r* = 0.58–0.67.^38^

QoL was measured using the parent-report version of the Inventar zur Erfassung der Lebensqualität bei Kindern und Jugendlichen for children and adolescents aged 6–18 years.^39^ In the ILK, QoL is divided into six different domains. Each of the six domains is covered by one question. The six domains are the following: school, family, social contacts with peers, interests and leisure activities, physical health, and mental health. Answers are given on a 5-point Likert scale (very good, rather good, partly good, rather poor, very poor). An internal reliability of α = 0.55–0.76 and a test–retest reliability of *r* = 0.60–0.80 (test interval = 2–6 weeks) has been presented.^39^ Out of the six domains, a QoL score and a problem score were calculated according to the manual. For interpretation, absolute scores were transformed into percentile ranks. The interpretation for the QoL score is as follows: ≤15 = below average QoL; 16–84 = average QoL; ≥85 = above average QoL. The interpretation for the problem score is as follows: ≥75 = possible problems; <75 = no problems. Whenever possible, all questionnaires were filled out before the neuropsychological assessment.

### Statistical analysis

IBM SPSS 25.0 was used for statistical analysis (Armonk, New York). For data visualization, Microsoft Excel (2016; https://office.microsoft.com) was used. A *p* value of < 0.05 was set as significant (two-sided). Cohen’s d was computed to estimate effect size small effect size = 0.1, medium effect size = 0.3, large effect size = 0.5.^40^ The normality of the data was examined with the Shapiro-Wilk test.

To compare cognitive performance, psychosocial functions, and parent-rated QoL between T1 and T2, one-sample t-tests or Wilcoxon tests were conducted. Change scores (absolute differences scores; absΔ) were calculated for processing speed, working memory, psychosocial functions, and QoL using the formula T2 – T1. We refrain from reporting relative difference as cognitive performance at T1 is a variable of interest and should not be partialled out of the analysis. Based on the change scores (absΔ), patients were divided into a stable or favorable group (stable or increase of standard scores) and a non-favorable group (any decrease of standard scores) for processing speed, working memory, psychosocial functions, and QoL. The relationship between working memory, processing speed, psychosocial, and QoL change scores (absΔ) was examined with a Spearman correlation. The stable or favorable and the non-favorable cognitive development group was compared in respect to nonverbal intelligence at T1 using a two-sample *t* test.

Multiple linear regression analyses were conducted to examine the relation between various potential risk factors and cognitive as well as psychosocial functions. Separate regression models were applied for each of the dependent variables: working memory, processing speed, and psychosocial functions at T2. Initially, each model included age at T1, sex, CNS involvement, cancer type, and treatment modality as independent variables. However, due to high multi-collinearity between CNS involvement and cancer type, indicated by variance inflation factors (VIF) ranging from 7.14 to 17.69 across the different models for each respective dependent variable, cancer type was removed from the models. Cancer type was initially represented by three dummy variables, which contributed to the complexity of the model. Given our relatively small sample sizes, ranging from 31 to 54 depending on the dependent variable, we opted for a simpler model that includes only CNS involvement, a dichotomous variable, along with the other predictors. Finally, each model included age at T1, sex, CNS involvement, and treatment modality as independent variables, controlling for measures at T1 of the respective dependent variables to account for initial cognitive or psychosocial functions. To address potential issues of heteroskedasticity, we employed heteroskedasticity-consistent standard errors (HC3) across all models. This decision was based on the recommendation by Long and Ervin (2000),^41^ who suggested that HC3 could be used reliably regardless of the presence or absence of heteroskedasticity, as evidenced by simulations.^42^ We did not adjust for multiple testing as our analysis were exploratory.^43^

## Results

Demographic and medical data are shown in Table 1.

**Table 1:** Demographic and medical data.

Demographics	*n*	*M (SD)* Range
Non-verbal intelligence at T1	44	109.09 (16.05)
		83–142
Age at T1 (y)	57	9.44 (2.92)
		4.02–14.41
Sex (m/f)	57	25/32
Mean time between T1 and T2 (y)	57	1.92 (0.39)
		1.08–3.10
CNS involvement (yes/no)^a^	57	20/37

Cancer Type	*n*	%
Brain tumor	18	31.6
Leukemia	19	33.33
Lymphoma	10	17.5
Other cases^b^	10	17.5

Treatment modality	*n*	%
Chemotherapy only	26	45.6
Surgery only	15	26.3
Combination^c^	15	26.3
No treatment	1	1.8

*n* number of patients, *M* mean, *SD* standard deviation, % incidence, *y* years, *T1* first two weeks of cancer treatment, *T2* one year after termination of cancer treatment;

^a^CNS involvement = tumor within the central nervous system;

^b^Ewing sarcoma, Neuroblastoma, Paraganglioma, Rhabdomyosarkoma, Sinonasal Glomangiopericytoma, localized pPNET frontobasal, Hepatoblastoma, Langerhans cell histiocytosis ;

^c^Surgery + Chemotherapy, Surgery + Radiotherapy, Chemotherapy + Radiotherapy, Surgery + Chemotherapy + Radiotherapy.

### Working memory and processing speed

Mean performance of working memory and processing speed were within the normal range at both time points and did not change significantly between T1 and T2 (see Table 2). Processing speed was below the normative range (<85) at T1 in *n* = 8 (14.8%) and at T2 in *n* = 7 (13.0%) of patients. Working memory was below the normative range at T1 as well as T2 in *n* = 5 (12.2%) of patients.

**Table 2:** Processing speed, working memory, psychosocial functions, and quality of life at T1, T2, and their change scores.

		T1 *M (SD)*	T2 *M (SD)*	*t/Z*	*p*	*d*	Change Score *M (SD)*
	*n*	Range	Range				Range
**Total group**							
*Cognition*							
Processing speed	54	103.44 (16.96)	101.91 (13.84)	0.64	0.520	0.09	−1.54 (17.56)
		65–147	71–134				−67–30
Working memory	41	99.61 (11.66)	98.05 (11.84)	0.82	0.420	0.13	−1.56 (12.25)
		70–126	77–129				−33–30
*Psychosocial functions*							
Total difficulties score^a^	31	7.42 (3.41)	7.55 (3.85)	−0.61	0.542	0.04	0.13 (3.50)
		2–15	2–15				−9–5
Emotional distress^a^	31	2.23 (1.28)	1.77 (1.61)	−1.66	0.097	0.31	−0.45 (1.48)
		0–6	0–6				−3–3
Conduct problems^a^	31	1.16 (0.97)	1.48 (1.34)	−1.18	0.238	0.22	0.32 (1.459)
		0–3	0–5				−3–3
Hyperactivity^a^	31	2.65 (2.12)	2.81 (2.09)	−0.61	0.539	0.11	0.16 (1.51)
		0–7	0–7				−3–3
Peer problems^a^	31	1.39 (1.61)	1.48 (1.67)	−0.37	0.711	0.07	0.10 (1.42)
		0–5	0–6				−3–3
Prosocial behavior ^b^	31	8.94 (1.06)	8.32 (1.54)	−2.33	0.020	0.36	−0.61 (1.69)
		5–10	4–10				−4–5
*QoL*							
Quality of life score^b^	21	54.45 (33.46)	56.78 (31.61)	−0.43	0.664	0.07	2.33 (32.95)
		0.0–97.3	4.0–100.0				−47.60–92.10
Problem score^a^	21	67.32 (25.72)	57.31 (25.03)	−1.53	0.133	0.36	−10.02 (28.13)
		0–100	27.2–100				−58.40–39.70
**Favorable development**							
*Cognition*							
Processing speed	28	95.64 (14.52)	107.11 (12.49)	−7.85	<0.001	1.48	11.46 (7.72)
		65–123	86–134				0–30
Working memory	17	95.29 (12.17)	105.06 (12.61)	−4.71	<0.001	1.14	9.76 (8.54)
		70–123	79–129				0–30
*Psychosocial functions*							
Total difficulties score^a^	17	8.06 (3.72)	5.88 (3.60)	−2.67	0.008	0.73	−2.18 (2.98)
		2–15	1–15				−9–0
Emotional distress^a^	24	2.50 (1.29)	1.50 (1.64)	−3.23	0.001	0.88	−1 (1.14)
		0–6	0–6				−3–0
Conduct problems^a^	19	1.37 (1.12)	0.79 (0.86)	−2.64	0.008	0.69	−0.58 (0.84)
		0–3	0–3				−3–0
Hyperactivity^a^	20	2.85 (2.18)	2.15 (2.03)	−2.56	0.011	0.68	−0.7 (1.03)
		0–7	0–6				−3–0
Peer problems^a^	21	1.52 (1.69)	0.86 (1.39)	−2.72	0.006	0.73	−0.67 (0.91)
		0–5	0–5				−3–0
Prosocial behavior^b^	16	8.81 (1.28)	9.38 (0.72)	−2.12	0.034	0.45	0.56 (1.26)
		5–10	8–10				0–5
*QoL*							
Quality of life score^b^	9	49.59 (36.14)	79.46 (21.31)	−2.67	0.008	0.96	29.87 (31.26)
		0.0–94.0	42.1–100.0				1.50–92.10
Problem score^a^	13	72.33 (19.97)	47.35 (20.26)	−2.805	0.005	1.07	−24.98 (23.44)
		47.6–100	27.2–95.4				−58.40–0
**Non-favorable development**							
*Cognition*							
Processing speed	26	111.85 (15.51)	96.31 (13.22)	5.66	<0.001	1.11	−15.54 (14.00)
		86–147	71–120				−67 to −3
Working memory	24	102.67 (10.48)	93.08 (8.43)	6.67	<0.001	1.14	−9.58 (6.96)
		84–126	77–111				−33 to −3
*Psychosocial functions*							
Total difficulties score^a^	14	6.64 (2.95)	9.57 (3.20)	−3.31	<0.001	2.12	2.93 (1.38)
		2–11	3–15				1–5
Emotional distress^a^	7	1.29 (0.76)	2.71 (1.11)	−2.46	0.014	1.82	1.43 (0.79)
		0–2	1–4				1–3
Conduct problems^a^	12	0.83 (0.58)	2.58 (1.24)	−3.14	0.002	1.81	1.75 (0.97)
		0–2	1–5				1–3
Hyperactivity^a^	11	2.27 (2.05)	4.00 (1.67)	−2.98	0.003	2.20	1.73 (0.79)
		0–6	2–7				1–3
Peer problems^a^	10	1.10 (1.45)	2.80 (1.48)	−2.85	0.004	2.07	1.70 (0.82)
		0–4	1–6				1–3
Prosocial behavior^b^	15	9.07 (0.80)	7.20 (1.37)	−3.46	<0.001	1.76	−1.87 (1.06)
		8–10	4–9				−4 to −1
*QoL*							
Quality of life score^b^	12	58.09 (32.43)	39.78 (27.38)	−3.06	0.002	1.37	−18.32 (13.33)
		9.2–97.3	4.0–85.9				−47.60 to −2.20
Problem score^a^	8	59.19 (32.92)	73.49 (24.54)	−2.69	0.031	0.95	14.30 (15.05)
		0–96	36–100				3–39.70

*n* sample size, *M* mean, *SD* standard deviation, *T1* first two weeks of cancer treatment, *T2* one year after termination of cancer treatment, *t* one-sample t-test, *Z* Wilcoxon test, *p* significance (α < 0.05), *d* Cohen’s d.

^a^higher scores indicate more problems.

^b^higher scores indicate less problems.

**Fig. 1:**
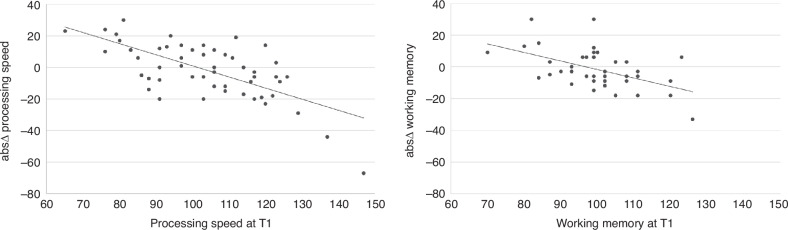
Correlation between performance at T1 and absolute cognitive change scores (abs∆). The first figure (on the left side) shows the correlation between the performance at T1 and the absolute change score for processing speed (*r* = −0.679, *p* < 0.001). The second figure (on the right side) shows the correlation between the performance at T1 and the absolute change score for working memory (*r* = −0.511, *p* < 0.001).

Around half of the patients showed a *stable or favorable cognitive development*, defined as cognitive performance remaining stable or increasing between T1 and T2 (PS: 51.9% of patients, *n* = 28/54; working memory: 41.4% of patients, *n* = 17/41). In patients with stable or favorable cognitive development, mean group performance increased significantly between T1 and T2 for processing speed and working memory (Table 2). The other half of the patients showed a *non-favorable cognitive development*, defined as cognitive performance decreasing between T1 and T2 (processing speed: 48.1% of patients, *n* = 26/54; working memory: 58.5% of patients, *n* = 24/41). In patients with a non-favorable cognitive development, mean group performance decreased significantly between T1 and T2 for processing speed and working memory (Table 2). For both processing speed and working memory, the stable or favorable and non-favorable groups did not differ in respect to nonverbal intelligence at T1 and risk factors such as age, sex, cancer type, and treatment modality. Furthermore, there was no association between processing speed and working memory change score (absΔ). Cognitive change scores (absΔ) were negatively associated with cognitive performance at T1, with worse cognitive performance at T1 relating to a stronger increase of working memory (*r* = −0.511, *p* < 0.001) and processing speed performance (*r* = −0.679, *p* < 0.001) and vice versa (Fig. 1).

### Psychosocial functions and quality of life

Mean psychosocial functions and QoL were within the normal range at both time points. At T1, 3.2 to 12.9% of all patients had borderline and 3.2–6.5% had abnormal scores in the different psychosocial subscales and the total difficulties score of the SDQ. At T2, 3.2–12.9% of patients had borderline and 3.2–6.5% had abnormal scores (*n*_total_ = 31). The mean prosocial behavior significantly improved between T1 and T2 (*Z* = −2.327, *p* = 0.020, *d* = 0.363).

The QoL score was below average in 14.3% of the patients at T1 as well as at T2. The problem score was below average in 47.6% of the patients at T1 and in 33.3% of the patients at T2 (*n*_total_ = 21). The mean QoL score remained stable between T1 and T2 (*Z* = −0.434, *p* = 0.664, *d* = 0.07) whereas the problem score decreased slightly (*Z* = 1.503, *p* = 0.133, *d* = 0.36), indicating a non-significant improvement in QoL. In *psychosocial functions*, stable or favorable change scores (stable or increased psychosocial functions) in the different domains occurred in 51.6–77.4% of patients (*n* = 16 to 24/31), whereas non-favorable development (decrease of psychosocial functions) occurred in 22.6–48.4% of patients (*n* = 7 to 15/31).

In QoL, a stable or favorable QoL change scores (absΔ) occurred in 42.9% (*n* = 9/21), whereas a non-favorable QoL change was presented in 54.1% of patients (*n* = 12/21). A stable or favorable change within the problem score (absΔ) occurred in 61.9% (*n* = 13/21), whereas a non-favorable change emerged in 38.1% (*n* = 8/21) of all patients.

### Correlation between cognitive and psychosocial functions

The processing speed change score was significantly correlated with the prosocial behavior change score (absΔ; *r* = 0.472, *p* = 0.010), indicating that an improvement in processing speed is related to an improvement in prosocial behavior. Neither changes in the SDQ total difficulties score, other SDQ subscales, nor the QoL score or problem score were associated with the development of processing speed or working memory between T1 and T2.

### Risk factors

Results from the regression analyses are summarized in Table 3. Multiple regressions regarding cognition revealed that age at T1 significantly predicts processing speed at T2 (*T* = 2.41, *p* = 0.020) with older age at T1 relating to better processing speed at T2. Regarding psychosocial functions, sex significantly predicts peer relations at T2 (*T* = 2.10, *p* = 0.046) meaning that male sex related to greater peer problems at T2. However, involvement of the CNS and treatment modality did not affect changes in cognition or psychosocial functions.

**Table 3:** Significant results of the multiple linear regression models for cognitive (3a) and psychosocial functions (3b) as dependent variables.

(3a) Processing speed at T2 (*n* = 54)					
Predictor	Reference category	*B (95% CI)*	*SE*	*T*	*p*
Intercept	–	50.73 (20.30, 81.17)	15.12	3.36	0.002*
Age at T1	–	1.61 (0.26, 2.96)	0.67	2.41	0.020*
Sex: Male	Female	5.78 (−2.06, 13.62)	3.89	1.49	0.144
CNS involvement of cancer: Yes	No	−4.34 (−16.15, 7.48)	5.87	−0.74	0.464
Treatment modality:	Chemotherapy only	1.77 (−8.76, 12.30)	5.23	0.34	0.736
Surgery only					
Treatment modality:	Chemotherapy only	−1.39 (−11.74, 8.97)	5.14	−0.27	0.788
Combination^a^					
Processing speed at T1	–	0.34 (0.07, 0.61)	0.13	2.51	0.016*

(3b) Peer problems at T2^b^ (*n* = 31)					
Predictor	Reference category	*B (95% CI)*	*SE*	*T*	*p*
Intercept	–	−0.22 (−2.37, 1.94)	1.04	−0.21	0.838
Age at T1	–	0.06 (−0.23, 0.35)	0.14	0.42	0.676
Sex: Male	Female	1.21 (0.02, 2.39)	0.57	2.10	0.046*
CNS involvement of cancer: Yes	No	−0.21 (−1.58, 1.16)	0.66	−0.32	0.754
Treatment modality:	Chemotherapy only	−0.14 (−1.31, 1.03)	0.57	−0.25	0.809
Surgery only					
Treatment modality:	Chemotherapy only	−0.58 (−1.87, 0.72)	0.63	−0.92	0.366
Combination^c^					
Peer problems at T1^a^	–	0.58 (0.23, 0.94)	0.17	3.39	0.002*

*B* unstandardized regression coefficient, *CI* confidence interval, *SE* heteroskedasticity-consistent standard error (HC3), *T* t-test, *p* level of statistical significance. *p < 0.05.

^a^Combination: Surgery + Chemotherapy, Surgery + Radiotherapy, Chemotherapy + Radiotherapy, Surgery + Chemotherapy + Radiotherapy.

^b^Score ranges between 0 and 10, higher scores indicate greater peer problems.

^c^Combination: Surgery + Chemotherapy, Surgery + Radiotherapy, Chemotherapy + Radiotherapy, Surgery + Chemotherapy + Radiotherapy.

## Discussion

The present study examined the development of working memory, processing speed, psychosocial functions and QoL of patients with pediatric cancer between the beginning of cancer treatment and one year after termination of cancer treatment. Further, associations between cognitive development and changes in psychosocial functioning and possible risk factors such as age at assessment, sex, cancer type, and treatment modality, were examined. Our results suggest that patients after pediatric cancer show individual developmental patterns for cognitive and psychosocial functions and QoL - despite test scores within the average range.

In detail, around half of the patients showed a stable or favorable cognitive development (stable or increased cognitive functions) whereas the other half of the patients showed a decrease in cognitive functions between T1 and T2. This result is in accordance with a recent study showing that more than half of the pediatric cancer survivors had age-appropriate cognitive functions in the long-term.^7^ Our data suggest, that individual factors such as age at diagnosis and sex play a role in the recovery process after cancer and its treatment and result in stable or favorable (restoration, substitution), neutral (no change) or non-favorable (pathological consequences) development. It is therefore of crucial importance to monitor the development of working memory and processing speed individually to support children with a non-favorable development as early and as well as possible.

Interestingly, in our patients, a significantly stronger cognitive increase was observed in patients with low cognitive performance at the beginning of treatment (T1). We cannot rule out the notion of a regression to the mean, whereby patients with worse cognitive performance at diagnosis might normalize their performance throughout rehabilitation. This statistical phenomenon could partially explain the observed cognitive increase in those with initially low performance and the decreases in those with initially high performance (see Fig. 1).

In respect to psychosocial functions, more than half of the patients showed a stable or favorable development, being in accordance with a study that showed that the majority of cancer survivors do not have an increase in psychological symptoms after cancer and its treatment.^44^ Further, our data suggest that mean prosocial behavior even improves between the beginning of treatment and one year after termination of treatment. A previous study proposes that prosocial behavior can be higher in cancer survivors than in a norm sample.^35^ This accentuation of prosocial behavior might be explained by post-traumatic growth that comes with increased focus on positive aspects of life.^45^ The correlation between the increase in prosocial behavior and the increase in processing speed presented in our results is corresponding with a recent study suggesting that in patients with brain tumor, processing speed played an important role in preventing psychological distress.^46^ While the directionality of a causal relationship is yet to be determined, research in both clinical and nonclinical samples has consistently demonstrated a relationship between executive functioning (i.e., processing speed) and prosocial behavior (see ref. ^47^ for a review). Executive functions have been implicated as potential moderators, mediators, and outcome measures in interventions to promote social competence.^47^ Hence, there might be a close link between processing speed and long-term prosocial behavior.

In more than half of the 21 patients with QoL assessments, a non-favorable development of parent-rated QoL was observed. Factors like parental distress and psychosocial family risk have been shown to influence the parent rating of QoL.^48^ However, at T1 as well as T2, the majority (namely 85.7%) of the parents reported a good QoL of their children. Hence, our data coincide with other studies showing that most pediatric cancer survivors present QoL scores within the normative range.^49^

A multiple linear regression model revealed that age at T1 – representative of age at diagnosis – significantly predicted processing speed at T2, supporting existing evidence that identifies age at diagnosis as a risk factor for cognitive problems in pediatric cancer patients. Younger children might be particularly vulnerable to the cancer-related long-term sequelae due to the rapid cerebral development occurring in early childhood. Cancer and its treatment partially interrupts these important developmental processes.^5,15,50^ Additionally, the regression analyses indicated that male sex predicted peer problems at T2. Female sex has traditionally been considered a risk factor for cognitive problems in pediatric cancer patients. However, recent studies have depicted a more nuanced view of sex as a risk factor. While in certain cancer populations female sex is still identified as a risk factor,^51,52^ other research suggests that cognitive functions in female patients improve more strongly over time compared to male patients.^53^ The findings on sex as a risk factor for cognitive problems might also apply to psychosocial functions, given their close relationship to cognitive functions.^20^

The present study has several potential limitations. T1 data was collected within the first two weeks of cancer treatment. This is an assessment in the very early stage after pediatric cancer. Some patients likely felt sick due to disease and intense treatments, and the parents and families suffered from high distress. Both might influence their responses to neuropsychological assessments and questionnaires. Overall, there is a large likelihood for a selection bias: Patients feeling too sick for a neuropsychological assessment shortly after diagnosis were likely missed at T1 and patients feeling well one year after cancer diagnosis were likely missed at T2 (as they did not see a need for an additional neuropsychological follow-up). Some patients did not agree to the general consent and hence their results were not included in the present data, rendering the presentation of data of non-participants impossible. Furthermore, it is a retrospective clinical record review and hence, the assessments were organized in the clinical routine, in a single center by different clinicians including a heterogeneous group of patients with different cancer types. Missing values are due to the fact that not all patients were followed-up in the clinical routine in a standardized manner. Another limitation of this study is that the patient group is skewed towards higher nonverbal intelligence at baseline. Last but not least, due to the relatively small sample size, regression analysis varied in respect to sample sizes and have to be interpreted with caution. All these issues must be considered when reflecting on the generalizability of the results.

Despite these limitations, this retrospective study is valuable as it reflects the clinical reality of our patient sample and reinforces existing findings from the literature. The variability in cognitive development observed in our sample (favorable vs. non-favorable outcome) highlights the importance of individual neuropsychological monitoring and interventions. Furthermore, the close link between psychosocial and cognitive functions underscores the opportunity and need to foster not only cognitive functioning but also psychosocial competence and interactions after pediatric cancer in the long-term.^15^ Pediatric patients with cancer often experience social isolation during their hospital stays. By integrating cognitive and psychosocial interventions, we can better support our patients in overcoming multifaceted challenges they encounter during and after cancer and its treatment.^54^ Future studies could focus on a longer observation periods after pediatric cancer, including additional cognitive variables such as, i.e., executive functions or attention skills and recruiting a larger sample size that would enable the thorough investigation of risk and protective factors.

To conclude, pediatric cancer patients display a wide variability in respect to their development of working memory, processing speed, psychosocial functioning, and QoL with about half of the patients showing stable or favorable whereas the other half experiencing non-favorable cognitive development. The observed variability of developmental pathways highlight the importance of early individualized patient monitoring and the supply of interventions strengthening cognition and psychosocial functions right after diagnosis (i.e., cognitive training, strategy training, metacognitive training, psychotherapy, physical training).

## References

[1] Phillips, N. S. et al. Late-onset cognitive impairment and modifiable risk factors in adult childhood cancer survivors. *JAMA Netw. Open* **6**, E2316077 (2023). 37256617 10.1001/jamanetworkopen.2023.16077 PMC10233416

[2] Schultze-Lutter, F., Schimmelmann, B. G. & Schmidt, S. J. Resilience, risk, mental health and well-being: associations and conceptual differences. *Eur. Child Adolesc. Psychiatry* **25**, 459–466 (2016). 27105994 10.1007/s00787-016-0851-4

[3] Siegwart, V., Schürch, K., Benzing, V., Roessler, J. & Everts, R. Personal and social resources are linked to cognition and health-related quality of life in childhood cancer survivors. *Children* **9**, 936 (2022). 35883920 10.3390/children9070936 PMC9322872

[4] Hardy, S. J., Krull, K. R., Wefel, J. S., & Janelsins, M. Cognitive changes in cancer survivors. *Am. Soc. Clin. Oncol. Educ. Book*, **38**10.1200/edbk_201179 (2018). 10.1200/EDBK_201179 30231372

[5] Krull, K. R., Hardy, K. K., Kahalley, L. S., Schuitema, I. & Kesler, S. R. Neurocognitive outcomes and interventions in long-term survivors of childhood cancer. *J. Clin. Oncol.* **36**, 2181–2189 (2018). 29874137 10.1200/JCO.2017.76.4696 PMC6553837

[6] Moore, B. D. Neurocognitive outcomes in survivors of childhood cancer. *J. Pediatr. Psychol.* **30**, 51–63 (2005). 15610985 10.1093/jpepsy/jsi016

[7] Siegwart, V. et al. Cognition, psychosocial functioning, and health-related quality of life among childhood cancer survivors. *Neuropsychol. Rehabil.* **32**, 922–945 (2020). 33208044 10.1080/09602011.2020.1844243

[8] Trapani, J. A., & Murdaugh, D. L. Processing efficiency in pediatric cancer survivors: a review and operationalization for outcomes research and clinical utility. In *Brain and Behavior* **12**, 10.1002/brb3.2809 (2022). 10.1002/brb3.2809 PMC9759139 36330565

[9] Morrison, A. B. & Chein, J. M. Does working memory training work? The promise and challenges of enhancing cognition by training working memory. *Psychonomic Bull. Rev.* **18**, 46–60 (2011). 10.3758/s13423-010-0034-0 21327348

[10] Kail, R. Speed of information processing: developmental change and links to intelligence. *J. Sch. Psychol.* **38**, 51–61 (2000).

[11] Reichenberg, A. & Harvey, P. D. Neuropsychological impairments in schizophrenia: integration of performance-based and brain imaging findings. *Psychol. Bull.* **133**, 833 (2007). 17723032 10.1037/0033-2909.133.5.833

[12] Mulhern, R. K., & Butler, R. W. Neurocognitive sequelae of childhood cancers and their treatment. *Pediatr. Rehabil*. 10.1080/13638490310001655528 (2004). 10.1080/13638490310001655528 14744668

[13] Hillary, F. G. et al. The nature of processing speed deficits in traumatic brain injury: is less brain more? *Brain Imaging Behav.* **4**, 141–154 (2010). 20502993 10.1007/s11682-010-9094-z

[14] Anderson, V., Spencer-Smith, M., & Wood, A. Do children really recover better? Neurobehavioural plasticity after early brain insult. *Brain* **134**, 2197–2221 (2011). 10.1093/brain/awr103 21784775

[15] Jones, R. M., & Pattwell, S. S. Future considerations for pediatric cancer survivorship: Translational perspectives from developmental neuroscience. *Dev. Cognit. Neurosci.* **38**10.1016/j.dcn.2019.100657 (2019). 10.1016/j.dcn.2019.100657 PMC6697051 31158802

[16] Schuerch, K. et al. How is cerebral perfusion associated with functional outcome in pediatric cancer survivors? *Dev. Neuropsychol.* **48**, 186–202 (2023). 37248710 10.1080/87565641.2023.2215360

[17] Aarsen, F. K. et al. Functional outcome after low-grade astrocytoma treatment in childhood. *Cancer* **106**, 396–402 (2005). 10.1002/cncr.21612 16353203

[18] Bitsko M. J. et al. Psychosocial late effects in pediatric cancer survivors: a report from the children’s oncology group. *Pediatr. Blood Cancer* **63**, 337–343 (2016). 10.1002/pbc.25773 PMC4715481 26488337

[19] Eilertsen, M. E. B., Rannestad, T., Indredavik, M. S. & Vik, T. Psychosocial health in children and adolescents surviving cancer. *Scand. J. Caring Sci.* **25**, 725–734 (2011). 21418265 10.1111/j.1471-6712.2011.00883.x

[20] Ehrhardt, M. J. et al. Neurocognitive, psychosocial, and quality-of-life outcomes in adult survivors of childhood non-hodgkin lymphoma. *Cancer* **124**, 417–425 (2018). 28915338 10.1002/cncr.31019 PMC5760296

[21] Andreotti, C., Root, J. C., Ahles, T. A., McEwen, B. S. & Compas, B. E. Cancer, coping, and cognition: a model for the role of stress reactivity in cancer-related cognitive decline. *Psycho-Oncol.* **24**, 617–623 (2015). 10.1002/pon.3683 PMC4387099 25286084

[22] Askins, M. A. & Moore, B. D.Preventing neurocognitive late effects in childhood cancer survivors. *J. Child Neurol.* **23**, 1160–1171 (2008). 18952582 10.1177/0883073808321065 PMC3674758

[23] Redmond, K. J. et al. Association of neuronal injury in the genu and body of corpus callosum following cranial irradiation in children with impaired cognitive control: a prospective study. *Int. J. Radiat. Oncol. Biol. Phys.* **101**, 1234–1242 (2018). 29908790 10.1016/j.ijrobp.2018.04.037 PMC6050077

[24] Elalfy, M., Ragab, I., Azab, I., Amin, S., & Abdel-Maguid, M. Neurocognitive outcome and white matter anisotropy in childhood acute lymphoblastic leukemia survivors treated with different protocols. *Pediatr. Hematol. Oncol.* **31,**10.3109/08880018.2013.871763 (2014). 10.3109/08880018.2013.871763 24498883

[25] Krull K. R. et al. Chemotherapy pharmacodynamics and neuroimaging and neurocognitive outcomes in long-term survivors of childhood acute lymphoblastic leukemia. *J. Clin. Oncol.* **34**, 10.1200/JCO.2015.65.4574 (2016). 10.1200/JCO.2015.65.4574 PMC5321052 27269941

[26] Margelisch K. et al. Cognitive dysfunction in children with brain tumors at diagnosis. *Pediatr. Blood Cancer* **62**, 10.1002/pbc.25596 (2015). 10.1002/pbc.25596 PMC5054885 26053691

[27] Walhovd, K. B., Howell, G. R., Ritchie, S. J., Staff, R. T. & Cotman, C. W. What are the earlier life contributions to reserve and resilience? *Neurobiol. Aging* **83**, 135–139 (2019). 31307838 10.1016/j.neurobiolaging.2019.04.014

[28] Windle, G. What is resilience? A review and concept analysis. *Rev. Clin. Gerontol.* **21**, 10.1017/S0959259810000420 (2011).

[29] Benzing V. et al. The Brainfit study: efficacy of cognitive training and exergaming in pediatric cancer survivors - a randomized controlled trial. *BMC Cancer* **18**, 10.1186/s12885-017-3933-x (2018). 10.1186/s12885-017-3933-x PMC5753470 29298678

[30] Benzing, V. et al. Effects of cognitive training and exergaming in pediatric cancer survivors - a randomized clinical trial. *Med. Sci. Sports Exerc.* **52**, 2293–2302 (2020). 33064404 10.1249/MSS.0000000000002386 PMC7556245

[31] Brown, L. *Test of Nonverbal Intelligence - Handbook of Nonverbal Assessment* (eds. R. S. McCallum) pp. 191–221. (Springer, 2003). 10.1007/978-1-4615-0153-4_10.

[32] Petermann, F., Ricken, G., Fritz, A., Schuck, K.-D., & Preuß, U. Wechsler preschool and primary scale – Third edition. *Deutschsprachige Adaption nach* D. Wechsler (3rd ed.). Pearson Assessment. (2014).

[33] Petermann, F., & Petermann, U. *Wechsler Intelligence Scale for Children - Fourth Edition. Manual 1: Grundlagen, Testauswertung und Interpretation. Übersetzung und Adaptation der WISC-IV von David Wechsler*. Pearson. (2011).

[34] Goodman, R. The strengths and difficulties questionnaire: a research note. *J. Child Psychol. Psychiatry* **38**, 581–586 (1997). 9255702 10.1111/j.1469-7610.1997.tb01545.x

[35] Mader, L. et al. Social, emotional, and behavioral functioning in young childhood cancer survivors with chronic health conditions. *Pediatr. Blood Cancer* **69**, 1–11 (2022). 10.1002/pbc.29756 35561093

[36] Woerner, W. et al. Normal values and evaluation of the German parents’ version of Strengths and DIfficulties Questionnaire (SDQ): results of a representative field study. *Z. Fur Kinder-Und Jugendpsychiatr. Und Psychother.* **30**, 105–112 (2002). 10.1024//1422-4917.30.2.105 12053874

[37] Woerner, W., Becker, A., & Rothenberger, A. Normative data and scale properties of the German parent SDQ. *Eur. Child Adolesc. Psychiatry, Supplement*, **13**, 10.1007/s00787-004-2002-6 (2004). 10.1007/s00787-004-2002-6 15243780

[38] Lohbeck, A., Schultheiß, J., Petermann, F. & Petermann, U. The German self-report version of the Strengths and Difficulties Questionnaire (SDQ-Deu-S): psychometric properties, factor structure, and critical values. *Diagnostica* **61**, 222–235 (2015).

[39] Mattejat, F., & Remschmidt, H. *ILK: Inventar zur Erfassung der Lebensqualität bei Kindern und Jugendlichen: Ratingbogen für Kinder, Jugendliche und Eltern*. Verlag Hans Huber. (2006).

[40] Cohen, J. Statistical power analysis. *Curr. Directions Psychol. Sci.* **1**, 98–101 (1992).

[41] Long, J. S., & Ervin, L. H. Using heteroscedasticity consistent standard errors in the linear regression model. *Am. Stat.* **54,**10.1080/00031305.2000.10474549 (2000).

[42] Hayes, A. F., & Cai, L. Using heteroskedasticity-consistent standard error estimators in OLS regression: an introduction and software implementation. *Behav. Res. Methods* **39**, 10.3758/BF03192961 (2007). 10.3758/bf03192961 18183883

[43] Althouse, A. D. Adjust for multiple comparisons? It’s not that simple. *Ann. Thorac. Surg.* **101**, 1644–1645 (2016). 27106412 10.1016/j.athoracsur.2015.11.024

[44] Brinkman, T. M. et al. Behavioral, social, and emotional symptom comorbidities and profiles in adolescent survivors of childhood cancer: a report from the childhood cancer survivor study. *J. Clin. Oncol.* **34**, 3417–3425 (2016). 27432919 10.1200/JCO.2016.66.4789 PMC5035122

[45] Barakat, L. P., Alderfer, M. A. & Kazak, A. E. Posttraumatic growth in adolescent survivors of cancer and their mothers and fathers. *J. Pediatr. Psychol.* **31**, 413–419 (2006). 16093518 10.1093/jpepsy/jsj058

[46] Oprandi M. C. et al. The influence of Socioeconomic Status (SES) and processing speed on the psychological adjustment and wellbeing of pediatric brain tumor survivors. *Cancers*, **14**, 10.3390/cancers14133075 (2022). 10.3390/cancers14133075 PMC9264789 35804846

[47] Riggs, N. R., Jahromi, L. B., Razza, R. P., Dillworth-Bart, J. E., & Mueller, U. Executive function and the promotion of social-emotional competence. *J. Appl. Dev. Psychol.* **27**, 10.1016/j.appdev.2006.04.002 (2006).

[48] Racine, N. M., Khu, M., Reynolds, K., Guilcher, G. M. T. & Schulte, F. S. M. Quality of life in pediatric cancer survivors: Contributions of parental distress and psychosocial family risk. *Curr. Oncol.* **25**, 41–48 (2018). 29507482 10.3747/co.25.3768 PMC5832275

[49] Zeltzer, L. K. et al. Psychosocial outcomes and health-related quality of life in adult childhood cancer survivors: a report from the childhood cancer survivor study. *Cancer Epidemiol. Biomark. Prev.* **17**, 435–446 (2008). 10.1158/1055-9965.EPI-07-2541 18268128

[50] Kahalley, L. S. et al. Slower processing speed after treatment for pediatric brain tumor and acute lymphoblastic leukemia. *Psycho-Oncol.* **22**, 1979–1986 (2013). 10.1002/pon.3255 PMC3740073 23447439

[51] Gandy K. et al. Sex-based differences in functional brain activity during working memory in survivors of pediatric acute lymphoblastic leukemia. *JNCI Cancer Spectrum*, **6**, 10.1093/jncics/pkac026 (2022). 10.1093/jncics/pkac026 PMC9041337 35603857

[52] van der Plas E. et al. Sex-Specific Associations between Chemotherapy, Chronic Conditions, and Neurocognitive Impairment in Acute Lymphoblastic Leukemia Survivors: a Report from the Childhood Cancer Survivor Study. *J. Natl Cancer Institute* **113**, 10.1093/jnci/djaa136 (2021). 10.1093/jnci/djaa136 PMC8096369 32882041

[53] Bledsoe J. C. et al. Differential trajectories of neurocognitive functioning in females versus males following treatment for pediatric brain tumors. *Neuro-Oncol.* **21**, 10.1093/neuonc/noz092 (2019). 10.1093/neuonc/noz092 PMC6784260 31123753

[54] Christiansen H. L. et al. Providing children and adolescents opportunities for social interaction as a standard of care in pediatric oncology. *Pediatr. Blood Cancer*, **62**, 10.1002/pbc.25774 (2015). 10.1002/pbc.25774 26700923

